# Effects of Kuan-Sin-Yin decoction on immunomodulation and tumorigenesis in mouse tumor models

**DOI:** 10.1186/1472-6882-14-488

**Published:** 2014-12-15

**Authors:** Tsai-Feng Li, Ching-Cheng Lin, Hui-ping Tsai, Chung-Hua Hsu, Shu-Ling Fu

**Affiliations:** Institute of Traditional Medicine, National Yang-Ming University, Taipei, 11221 Taiwan; Herbal Medicine Product Technology Division of Biomedical Technology and Device Research Laboratories, Industrial Technology Research Institute, Hsinchu, Taiwan

**Keywords:** Traditional Chinese medicine, Anti-cancer, Immunomodulation, Cytostatic effect, Kuan-Sin-Yin

## Abstract

**Background:**

Complementary therapies are widely used among cancer patients. Kuan-Sin-Yin (KSY) decoction, a popular qi-promoting herbal medicine, was constituted with several herbs known to exhibit immunomodulating or anticancer activity. After combining these herbs as a compound formula, it is necessary to reassess the immunomodulation effects, the effects on tumor growth, and possible toxicity of KSY.

**Methods:**

The anti-cancer effects of KSY *in vivo* were determined by measuring the tumor volumes, anticancer-associated cytokines (IFN-gamma, TNF-alpha, IL-2, and IL-12), accumulation of tumor infiltrating leukocytes (TILs), proliferation and apoptosis-related molecular markers (Ki-67, p53, p21, activated caspase 3, and cleaved PARP), and an *in situ* TUNEL assay. The body weight and serum chemistry of treated mice were also assessed. *In vitro*, the effects of KSY were evaluated using MTT assay, BrdU incorporation assay and cell growth curve.

**Results:**

*In vivo*, KSY suppressed bladder or lung cancer growth but did not promote the production of cytokines nor increase the accumulation of TILs. The expression of p53 and p21 in KSY-treated mice were increased. The numbers of apoptotic tumor cells and the expression of apoptosis marker proteins (Caspase 3 and cleaved PARP) were not significantly elevated after KSY treatment. *In vitro*, the viability and proliferation of tumor cells, but not normal cells, were suppressed by KSY treatment. No significant toxicity was found in KSY-treated mice.

**Conclusions:**

KSY suppressed the tumor growth *in vivo* and *in vitro*, which resulted from its cytostatic effects on cancer cells, rather than the induction of anti-cancer immunity. Under these experimental conditions, no apparent toxicity was observed.

**Electronic supplementary material:**

The online version of this article (doi:10.1186/1472-6882-14-488) contains supplementary material, which is available to authorized users.

## Background

Cancer is the leading cause of death worldwide. Tumor cells have developed a variety of strategies to survive and suppress anti-tumor immune response. The conventional treatments such as surgery, radiotherapy, and chemotherapy impair the patient’s immunity [1]. When the immune system is not working well, the frequent infections or chronic inflammation status might contribute to chronic fatigue and tumor progression or recurrence. On the other hand, micrometastases of cancer stem cells frequently lead to tumor relapse and therapeutic failure after completing the conventional treatment courses [2].

Within the cancer patient population, complementary and alternative medicine (CAM) is used widespread and estimated to be as high as 64% [3]. It was expected that CAM options could relieve the general illness or undesirable side effects, stimulate and protect immunity, or prevent further tumor progression or recurrence [4–7]. In Taiwan, the use of traditional Chinese medicine (TCM) has been reimbursed by the National Health Insurance since 1996. More than 60% of outpatients obtained from the National Health Insurance database (NHIRD) had used TCM from 1996-2001 [8]. TCM is very popular in Taiwan, including fighting the related symptoms of malignant neoplasm.

A compound herbal formula, Kuan-Sin-Yin (KSY) decoction had been used to “reinforce the healthy qi” in traditional Chinese medicine (TCM) theory and it was widely used to treat the dysfunctional gastrointestinal symptoms and chronic fatigue syndrome. In clinical trial study, KSY improved the score on the physical component of the World Health Organization Quality of Life-Brief Version (WHOQOL-BREF) of hepatitis B virus carriers [9]. Individual purified compounds and herbal extracts in KSY have been previously demonstrated to exhibit immunomodulatory or anti-cancer effects *in vivo* and *in vitro* [10–19]. *Astragalus membranaceus* and *Codonopsis pilosula*, which are ingredients of KSY, were reported that they could function as adjuvant therapies during chemotherapy and radiation therapy in clinical trials [1, 20–22].

Current observations indicate that KSY might function as an immunomodulator to suppress tumor growth and as a complementary cancer therapy. However, as a compound formula, these expected therapeutic effects of KSY are rarely studied. In this study, the effects of KSY on immunomodulation and tumorigenesis, as well its toxicity profile were examined in mouse tumor models.

## Methods

### Chemicals and reagents

3-(4,5-Dimethylthiazol-2-yl)-2,5-diphenyltetrazolium bromide (MTT), lipopolysaccharide (LPS), phytohemagglutinin (PHA), 4’,6-diamidino-2-phenylindole (DAPI), 5-bromo-2’-deoxyuridine (BrdU) were purchased from Sigma Chemical Co. (St. Louis, USA). Lipo-Dox (doxorubicin hydrochloride) was purchased from TTY Biopharm Co. (Taipei, Taiwan); OK-432 (Picibanil) was acquired from Chugai Pharmaceutical (Tokyo, Japan).

### Preparation of KSY

To prepare the KSY, concentrated aqueous extracts of seven Chinese herbs were mixed in fixed ratios (shown in Table 1) and boiled in 500 ml sterile water for 30 minutes. The aqueous extracts of the raw ingredients of KSY were provided by the manufacturer, Sun-Ten Pharmaceutical Co. Ltd. (Taipei, Taiwan). The amounts of heavy metals, residual pesticides and microbes in each herb were inspected and met the safety criteria of Taiwan Food and Drug Administration (TFDA). The final concentration was defined as the weight of the aqueous herbal mixtures (152.3 g) in 500 ml of ddH_2_O, which was approximately 0.3 g/ml. For *in vitro* experiments, a quantified amount (15 ml) of the KSY extract was processed with a freeze-dryer to obtain crystal powder (0.87 g), which was then re-dissolved in sterile ddH_2_O, filtered and stored as a stock solution (100 mg/ml) at -20°C. The protocol regarding HPLC analysis of KSY was described in the supplementary section “Additional file 1”.

**Table 1:** **Ingredients in KSY**

Medicinal plants	Portions used	Ratio
Dan Shen	Dried roots of *Codonopsis pilosula (Franch.)* Nannf.	2
Fu Ling	Dried sclerotium ofthe fungus*, Poria cocos (Schw.)* Wolf	2
Bai Zhu	Rhizome of *Atractylodes macrocephala* Koidez*.*	2
Gan Cao	Dried roots and rhizome of *Glycyrrhiza uralensis* Fisch.	1
Nu Zhen Zi	Dried fruits of *Ligustrum lucidum* Ait.	1
Huang Qi	Dried roots of *Astragalus membranaceus (Fisch.)* Bunge*.*	2
Guan huo xiang	Dried branches and leaves of *Pogostemon cablin (Blanco) Bench.*	2

### Cell lines and culture conditions

The mouse bladder carcinoma cell line MBT-2 (obtained from Dr. Lih-Hwa Hwang, National Yang-Ming University, Taiwan), the mouse Lewis lung carcinoma line LLC-1 (American Type Culture Collection; Manassas, USA) and the primary mouse embryo fibroblast MEF cell line (obtained from Dr. Ting-Feng Tsai, National Yang-Ming University, Taiwan) were cultured in Dulbecco’s modified Eagle medium (Invitrogen, CA, USA) supplemented with 10% heat-inactivated fetal bovine serum (FBS), 100 units/ml penicillin, 100 μg/ml streptomycin and 2 μM L-glutamine in a humidified 5% CO_2_ incubator at 37°C.

### Animals

C3H/HeN, BALB/cAnN-Foxn1nu/Cr1Nar1, and C57BL/6 mice were purchased from the National Laboratory Animal Center (Taipei, Taiwan) and maintained at the Animal Center of Yang-Ming University (Taipei, Taiwan) following the Guidelines for the Care and Use of Laboratory Animals (National Institutes of Health, USA). All experiments utilizing mice were approved by the Institutional Animal Care and Use Committee (IACUC Approval No. 1001246) at National Yang-Ming University (Taipei, Taiwan).

### Mouse tumor models and treatment protocols

**Figure 1:**
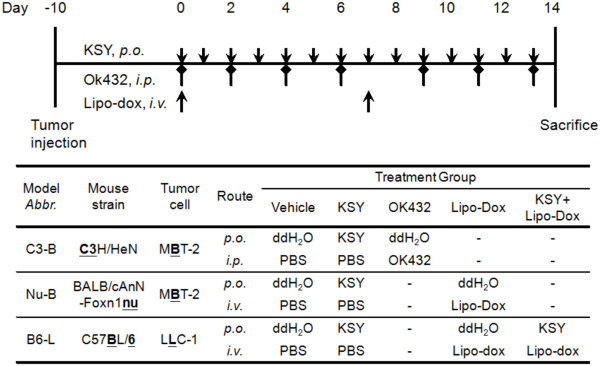
**Outline of tumor models and treatment protocols used in this study.** (Upper panel) treatment protocols and administration routes for different drugs. KSY: 1000 mg/kg/day, p.o.; OK-432: 4 mg/kg per injection, i.p.; Lipo-Dox: 1.5 mg/kg per injection, i.v.. (Bottom panel) tumor mouse models used in this study. In the C3-B model, C3H/HeN mice were injected with a syngeneic bladder cancer cell line MBT-2. In the Nu-B model, BALB/cAnN-Foxn1nu/Cr1Nar1 mice were inoculated with MBT-2 cells. In the B6-L model, C57BL/6 mice were injected with a syngeneic lung cancer cell line LLC-1. The detailed experimental procedures are described in the “Methods”.

Treatment protocols in three different mouse tumor models were used in this study, which are outlined in Figure 1. In all models, cancer cells (1 × 10^6^) were injected s.c. into the dorsum of mice. The tumor volume (*V*) was calculated by the following formula: *V* = 1/2(*L* × *W*^2^), where *L* is the length and *W* is the width of tumor. After tumor volumes reached 100 ± 20 mm^3^ (~10 days), mice were randomly assigned into different drug treatment groups (Figure 1, day 0).

For the bladder tumor model in immunocompetent mice (Figure 1, C3-B), MBT-2 bladder cancer cells were inoculated into C3H/HeN mice, and the mice were randomly divided into three different treatment groups: the KSY-treated group, the vehicle group and the OK432-treated group. OK432 is a *Streptococcal* immunotherapeutic agent used as the positive control. The KSY-treated group received KSY treatments (1000 mg/kg/day, p.o.). The OK432-treated group received OK-432 treatments (4 mg/kg, i.p.) on days 0, 2, 4, 6, 9, and 12.For the bladder tumor model in immunocompromised mice (Figure 1, Nu-B), the nude mouse strain BALB/cAnN-Foxn1nu/Cr1Nar1 were inoculated with MBT-2 bladder cancer cells and divided into three treatment groups: the KSY-treated group, the vehicle group and the liposome-doxorubicin (Lipo-Dox)-treated group. The cytotoxic chemotherapeutic drug liposome-doxorubicin was used as the positive control. The KSY-treated group received KSY (1000 mg/kg/day, p.o.). For the Lipo-Dox-treated group, the tumor-bearing mice were injected with Lipo-Dox (1.5 mg/kg in PBS i.v.) on days 0 and 7.

The vehicles for the KSY, OK-432, and Lipo-Dox groups were ddH_2_O, PBS, and PBS, respectively. The KSY dosage used in this study was referred from that used in clinical study described previously [9].

For the lung cancer model (Figure 1, B6-L) in immunocompetent mice, LLC-1 lung cancer cells were inoculated into the syngeneic C57BL/6 mouse strain. The experimental treatment protocol was similar to the Nu-B model protocol described above. For the co-treatment group combining KSY with Lipo-Dox, tumor-bearing mice were injected Lipo-Dox (1.5 mg/kg in PBS) *i.v.* on day 0 and 7, and at the same time, they were given KSY (1000 mg/kg/day) *p.o.* daily.

### Cytokine production measurements

The tumor tissues were lysed with protein extraction buffer (50 mM Tris-HCl, pH 7.4, 15 mM NaOH, 0.1% NP-40, 5 mM EDTA, and 10 mM EGTA) containing protease inhibitors (Sigma-Aldrich). The concentrations of cytokines in the tumor lysates (500 μg) were measured by ELISA kits (R&D Systems, Minneapolis, USA). Primary splenocytes were isolated from the treated mice as previously described [23], and then 3 × 10^6^ splenocytes from each treated mouse were cultured and stimulated with the T cell mitogen PHA for 72 h. Afterwards, the supernatants from the splenocytes were collected to measure the cytokine concentrations.

### Splenocyte-conditioned medium (SCM)-mediated cytotoxicity assay

The splenocytes (1.5 × 10^7^) were cultured for 48h with PHA and the culture medium was collected as splenocyte-conditioned medium (SCM). Next, MBT-2 cells (2 × 10^5^) were incubated with the SCM for an additional 48 h. The SCM-mediated cytotoxicity of MBT-2 cells was measured as previously described using propidium iodide (PI, 20 μg/ml) and a FACS Calibur flow cytometer (BD Biosciences, NJ, USA) [24].

### Quantitative Reverse Transcription Polymerase Chain Reaction (qRT-PCR)

The protocols for isolation of total RNA from tumor tissues and synthesis of cDNAs have been previously described [24]. The primer sequences used in this study are summarized in Additional file 2: Table S1). The GAPDH mRNA level was used as the internal control. All PCR reactions were performed using an Applied Biosystems model 7000 instrument (Applied Biosystems, California, USA).

### TUNEL assay

The apoptotic cells in paraffin-embedded tumor sections were detected with an *in situ* cell death detection kit based on the labeling of DNA strand breaks (Roche Diagnostics, Mannheim, Germany). The tumor sections were counterstained with DAPI and analyzed by fluorescence microscopy (Olympus BX61, Tokyo, Japan). The image processing and analysis were carried out using the MetaMorph^®^ Software (Molecular Devices, Inc., California, USA).

### Immunohistochemistry

Paraffin-embedded tumor sections (3 μm) were soaked in an antigen retrieval buffer containing 10 mM sodium citrate (pH 6.0) and treated twice with microwave irradiation (650 W) for 10 min. An antibody against Ki-67 (BD, California, USA) was incubated with the tumor sections overnight. After washing with PBS, the tumor sections were incubated with biotinylated secondary antibodies and an avidin/streptavidin-based detection system (Vectastain^®^ Elite^®^ ABC peroxidase kit, Vector laboratories, lnc., Burlingame, USA), followed by treatment with a 3, 3’-diaminobenzidine tetrahydrochloride (DAB) staining system (Merck Millipore, Billerica, MA, USA) and counterstaining with hematoxylin. The stained sections were imaged using a microscope (Olympus BX61, Tokyo, Japan).

### Immunoblotting

The tumor tissues were lysed with a protein extraction buffer containing protease inhibitors. Protein lysate (50 μg) from each sample was assessed for the expression of p53, p21, Caspase-3, PARP or GAPDH as previously described [25]. Band intensities were quantified using the Image J program, version 1.40 (NIH, Bethesda, USA).

### MTT assays

To measure cell viability, MBT-2 (3 × 10^3^), LLC-1 (1 × 10^3^) and MEF (3 × 10^3^) cells were cultured in 96-well plates overnight, treated with the vehicle (sterile ddH_2_O), KSY extract, or doxorubicin for 24, 48, and 72 h. Cellular metabolic activity was measured by MTT-based colorimetric assays as previously described [26].

### Growth curve and BrdU incorporation assay

To establish a growth curve, MBT-2 cells (1 × 10^5^) were cultured overnight and treated with vehicle (sterile ddH_2_O), KSY, or lipo-dox for 24, 48, and 72 h. Adherent and floating cells were both collected, and the number of viable cells was counted by trypan blue dye exclusion. For BrdU incorporation assays, MBT-2 cells (1 × 10^5^) were cultured overnight and synchronized by 24 h of growth in serum-free medium. The serum-starved cells were incubated with vehicle or drugs for 22 and 46 h and treated with 10 mΜ BrdU for 2 h, and then both the adherent and floating cells were collected. The harvested cells were fixed in 4% (w/v) buffered-formaldehyde in PBS, permeabilized with 0.5% Triton X-100 in PBS, and treated with DNase I. After incubating with an anti-BrdU antibody (BD, California, USA) and a fluorescein-conjugated secondary antibody (Jackson ImmunoResearch Laboratories, Inc., PA, USA), the cells were treated with RNase (20 μg/ml), stained with PI (20 μg/ml), and then analyzed by flow cytometry.

### Blood chemistry measurements

Blood samples were collected from mice under terminal anesthesia through cardiac punctures. Clear serum samples were prepared and measured with a Fuji Dri-Chem Clinical Chemistry Analyzer FDC 3500 (Norsted, Germany).

### Statistical analysis

All data are presented as the mean ± SEM (standard error of mean) and are from at least three independent experiments. The Mann-Whitney *U* test was used to determine the significance of the between-group differences. Statistical significance was set at *p* < 0.05. All *p* values were two-tailed, and all statistical analyses were performed with the SPSS statistics software (Statistical Product and Service Solutions, IBM, New York, USA).

## Results

The KSY dosage used in animal experiments is referred to the clinical dose for human subjects [9]. The human dosage of KSY was translated to mouse dosage (~1500 mg/kg) using a formula described previously [27]. After preliminary toxicity/efficacy experiments, the dosage of 1000 mg/kg was chosen for subsequent in vivo experiments.

### The HPLC analysis of KSY

The HPLC chromatogram of KSY was shown in Additional file 3: Figure S1 and marker compounds (glycyrrhizic acid, liquiritin, liquiritin apioside, and calycosin-7-O-β-D-glucoside) were well resolved by gradient elution. Analysis showed that 1 g KSY contained 29.59 ± 0.78 μg of calycosin-7-O- β-D-glucoside, 187.08 ± 4.28 μg of liquiritin apioside, 228.42 ± 2.91 μg of liquiritin, and 701.19 ± 7.92 μg of glycyrrhizic acid.

### KSY delays tumor growth in immunocompetent and immunocompromised mice

**Figure 2:**
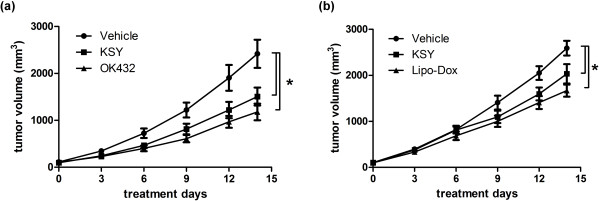
**KSY delays tumor growth in both immunocompetent and immunocompromised mice.** The MBT-2 cancer cells (1 × 10^6^) were injected s.c. into the dorsum of mice. After 10 days, tumor-bearing mice were randomly grouped and treated with vehicle or indicated drugs using the treatment protocol described in Figure 1. The mean tumor volumes of treated mice on the indicated days are shown. **(a)** The C3-B model (n = 17-20 for each group); **(b)** The Nu-B model (n = 14 for each group). *indicates p < 0.05 versus the vehicle group.

To investigate the effect of KSY treatment on tumor development, the growth of the MBT-2 bladder cancer cells in immunocompetent and immunocompromised hosts (Figure 1, C3-B and Nu-B, respectively) was examined. The tumor volumes of the KSY- and OK432-treated mice were reduced compared with the vehicle group in the C3-B model (Figure 2a). Similar suppressive effects were observed in the KSY- and Lipo-Dox-treated groups in the Nu-B model (Figure 2b).

### KSY does not induce anticancer immunity

**Figure 3:**
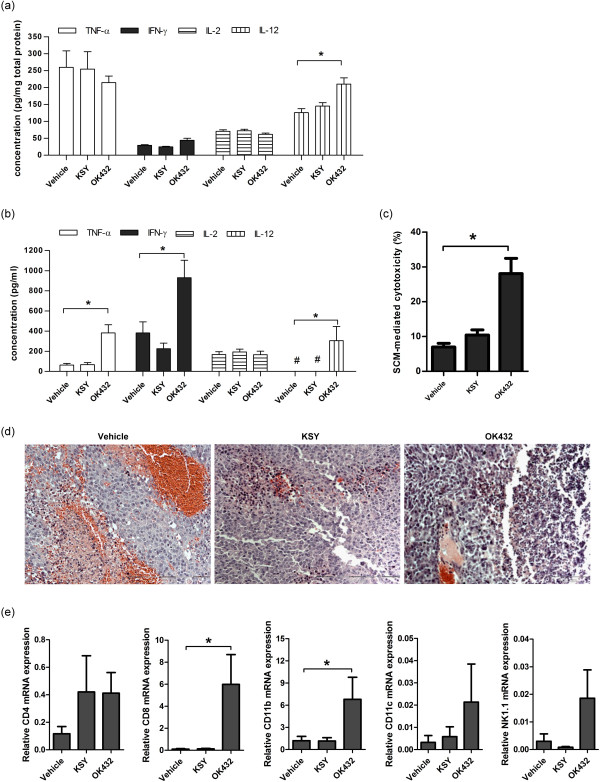
**KSY neither enhances the secretion of anticancer-associated cytokines, nor increases the recruitment of tumor-infiltrating leukocytes in tumor-bearing mice.** The anticancer immunity in the bladder cancer model (C3-B model in Figure 1) was evaluated. **(a)** The expressions of indicated cytokines in tumor tissues were measured by ELISA. **(b)** The splenocytes (3 × 10^6^/ml) from each treatment group were cultured in the presence of PHA (5 μg/ml) for 72 h, and the amount of cytokines in the culture medium were measured by ELISA. **(c)** The culture medium collected from the splenocytes of drug-treated mice (splenocyte-conditioned medium; SCM) was incubated with MBT-2 cells for 48 h. The death of MBT-2 cells was detected by PI stain and analyzed by flow cytometry. # indicates undetectable. *indicates p < 0.05 versus the vehicle group. n = 18-21. **(d)** Tumor tissues from each treatment group in C3-B model were fixed, sectioned and stained with hematoxylin and eosin (H&E stain) for microscopic examination. **(e)** The expression of various immune cell maker genes in tumor tissues of C3-B model mice was measured using qRT-PCR. CD4: T helper cells; CD8: cytotoxic T cells; CD11b: macrophages; CD11c: dendritic cells; NK1.1: NK cells. Three independent experiments were performed (n = 3 for each group). *indicates *p* < 0.05 versus the vehicle group.

In the C3-B mouse model, the expression of the anticancer-associated cytokines (TNF-α, IFN-γ, IL-2, and IL-12) were measured by ELISA assay in the host spleen and the local tumor area. The result showed that KSY did not increase the expression of these cytokines in the tumor area, as compared with the vehicle treatment (Figure 3a). Furthermore, the cytokine levels secreted from splenocytes isolated from KSY-treated mice, in the presence of PHA, were similar to that in vehicle groups (Figure 3b). Conversely, the TNF-α, IFN-γ and IL-12 secretions were increased in splenocytes of the OK-432 group (Figure 3b). The SCM-driven cytotoxicity of MBT-2 cells correlated with splenocyte cytokine expression, as the SCM-mediated cytotoxicity was significantly enhanced in the OK-432 group but not in the KSY group (Figure 3c).

The histological examination was next performed. The tumor-infiltrating leukocytes were significantly elevated in the tumors of OK-432-treated mice, but not in the KSY group and vehicle group (Figure 3d). Consequently, the amounts of tumor-infiltrating leukocytes after KSY treatment were determined by measuring of the mRNA expression levels of various immune cell markers. KSY treatment did not promote the recruitment of immune cells (i.e., CD4^+^ T cells, CD8^+^ T cells, dendritic cells, NK cells and macrophages) at tumor sites, but Ok-432 indeed elevated local immune cells (Figure 3e).

### KSY treatment reduces proliferation of cancer cell in vivo

**Figure 4:**
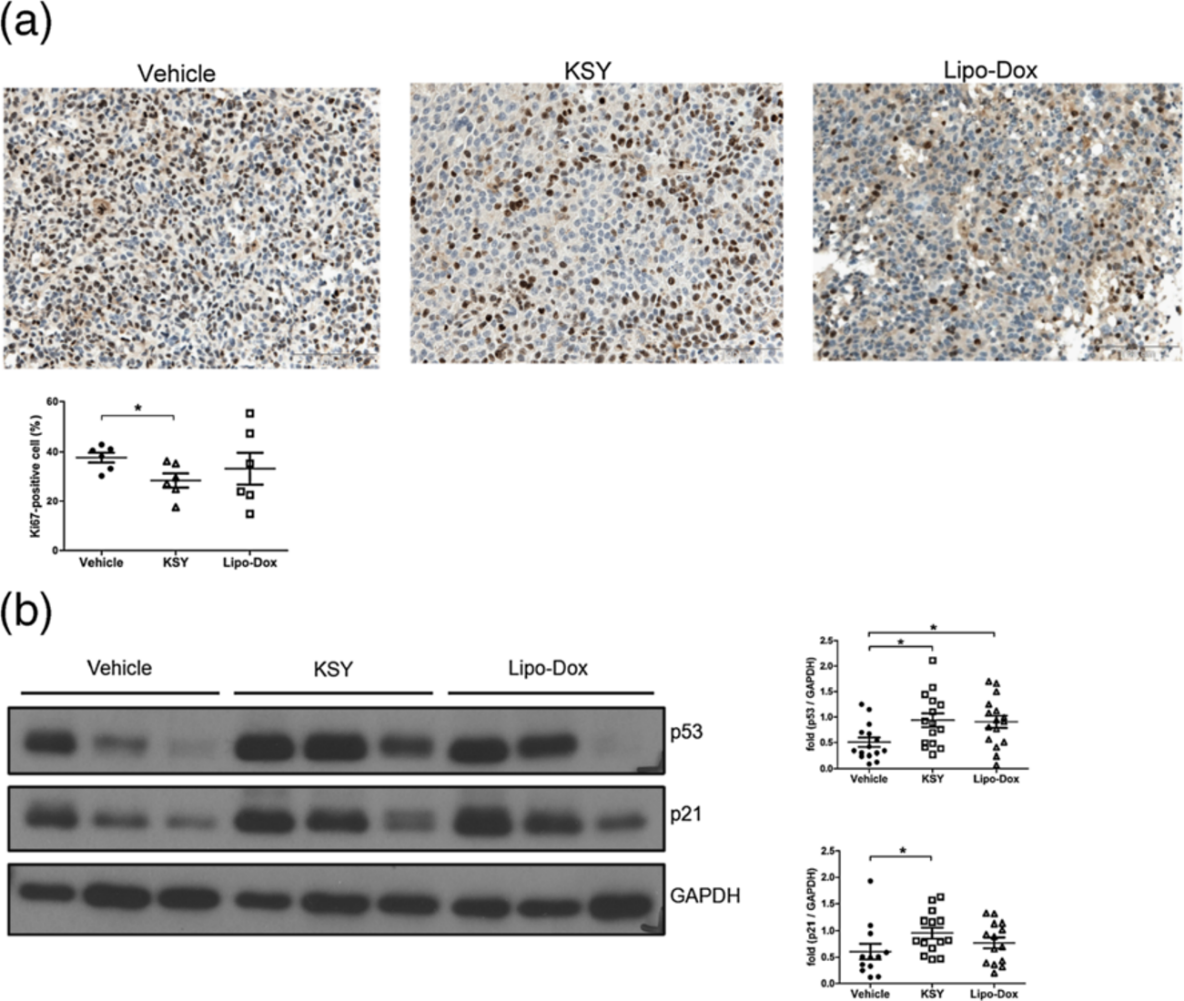
***In vivo*** **KSY treatment reduces Ki-67 expression and increases the expression of p53 and p21. (a)** In the Nu-B model, Ki-67-positive cells (brown) in tumors were detected by immunohistochemistry. Five fixed fields from each tumor section were analyzed for the number of Ki-67-positive cells. The percentage of Ki-67-positive cells was calculated by the number (Ki-67-positive cells/the total cell number of five fields) × 100%. The quantification of the percentage of Ki-67-positive cells is shown in the lower panel. **(b)** The protein expression levels ofp53 and p21 in tumors of Nu-B model were detected by Western blot. The quantification data are shown in the left panel. *indicates *p* < 0.05 versus the vehicle group.

The Ki-67 expression was measured by IHC in the tumor tissues in Nu-B mouse model. As shown in Figure 4a, the Ki-67 expression was significantly lower in both the Lipo-Dox- and KSY-treated groups compared with the vehicle group. Furthermore, the expression levels of cell cycle regulators, p53 and p21, in tumor tissues were elevated in the KSY group. The p53 expression of Lipo-Dox group was increased compared with the vehicle group (Figure 4b).

### KSY treatment does not increase tumor cell apoptosis in situ

**Figure 5:**
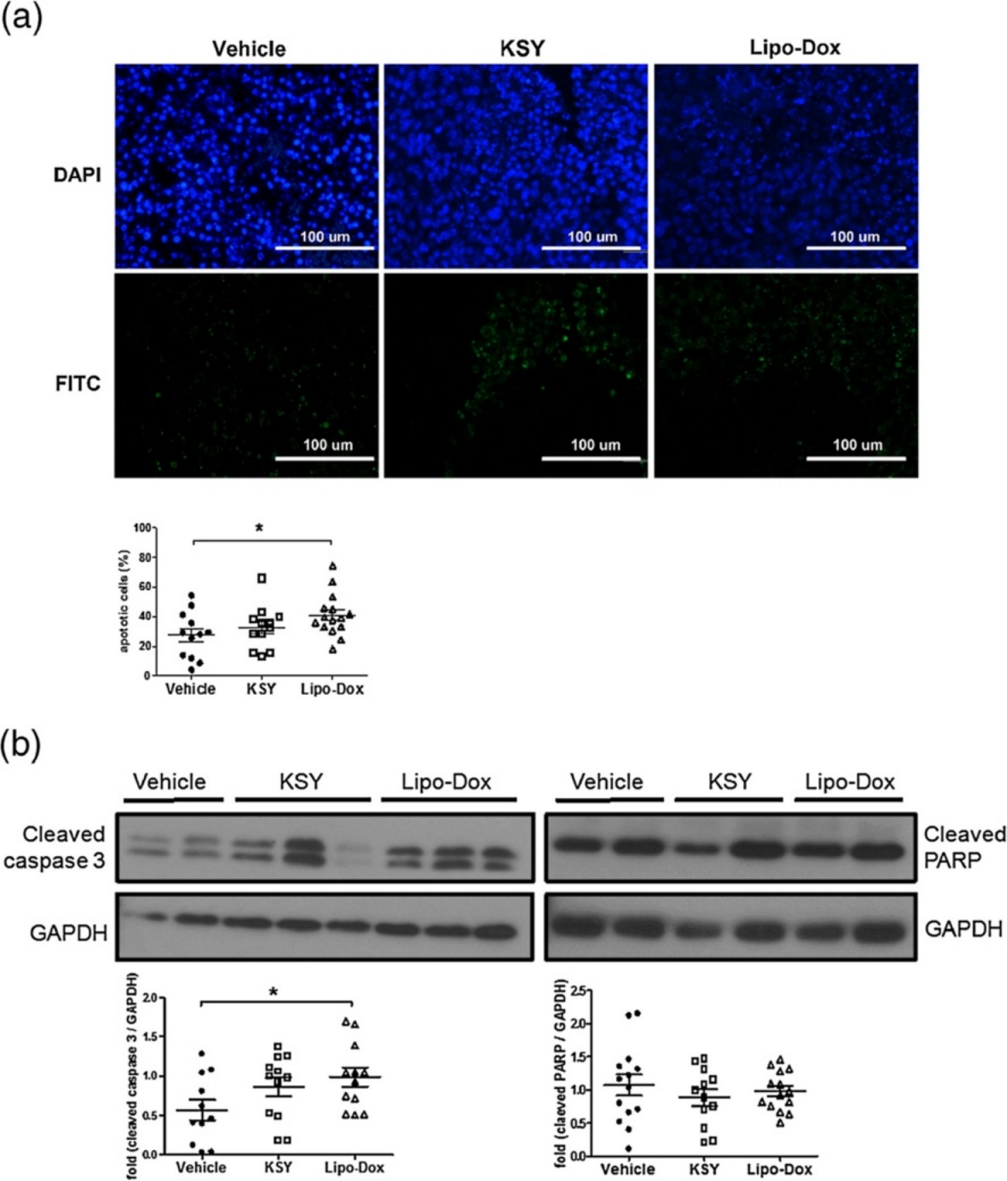
**KSY treatment does not increase tumor cell apoptosis. (a)** In the Nu-B model, the apoptotic cells (green) were detected by a TUNEL assay. Five independent fields from each tumor tissue section were analyzed for the numbers of TUNEL-positive cells. The percentage of apoptotic cells is shown in the bottom panel and was determined by the following equation: (the number of apoptotic cells/the total cell number of five fields) × 100%. **(b)** In the Nu-B model, the expression levels of active caspase 3 and cleaved PARP in tumor tissues were measured by Western blot. The quantification data are also shown. *indicates *p* < 0.05 versus the vehicle group.

The apoptotic tumor cells were visualized by an *in situ* TUNEL assay. KSY treatment did not enhance the tumor cell apoptosis (Figure 5a), and this observation was confirmed by the expression patterns of activated caspase 3 and cleaved PARP in the tumor tissues (Figure 5b).

### In vitro KSY treatment causes cytotoxicity on cancer cells but not on normal cells

**Figure 6:**
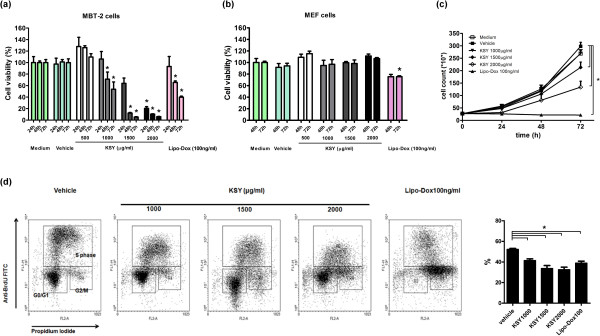
***In vitro*** **KSY treatment decreases cancer cell viability, suppresses proliferation, and interferes with cell cycle progression** ***.*** **(a)** The viability of MBT-2 cancer cells (3,000 cells/well in a 96-well plate) was determined by an MTT assay after a 24, 48, or 72 h vehicle or drug treatment at indicated concentrations. Doxorubicin (Lipo-Dox) served as the positive control. **(b)** An MTT assay was also used to measure the viability of primary MEF cells (3,000 cells/well in a 96-well plate). **(c)** MBT-2 cancer cells (1 × 10^5^) were treated with different concentrations of KSY for 24, 48 or 72 h, and the viable cells were determined using Trypan blue exclusion assays. Doxorubicin (Lipo-Dox) served as the positive control. **(d)** MBT-2 cells (1 × 10^5^) were treated with different concentrations of KSY for 24 h and then analyzed with BrdU/PI staining and flow cytometry. Representative FACS dot plots are shown in the left panel, and DNA synthesis (S phase) was determined by quantifying the BrdU-positive cells in the dot plot. Quantification data of BrdU-positive cells are shown in the right panel. *indicates *p* < 0.05 versus the vehicle group.

The cancer cell viability was examined by an MTT assay in vitro. As shown in Figure 6a, the KSY treatments reduced the viability of MBT-2 tumor cells in a concentration- and time-dependent manner. Under the same experimental conditions, KSY treatments did not cause significant cytotoxicity on primary mouse embryonic fibroblasts (MEFs), but doxorubicin treatment reduced MEF viability (Figure 6b).

### In vitro KSY treatment suppresses the proliferation of MBT-2 cells

As shown in Figure 6c, KSY treatment at 1500 μg/ml or 2000 μg/ml for 72 h significantly suppressed the proliferation of MBT-2 cells in a concentration-dependent manner. The DNA synthesis ability was further analyzed by BrdU/PI staining and flow cytometry. For MBT-2 cells subjected to a 24-h KSY treatment, the percentage of BrdU-positive cells (S phase) was significantly reduced (Figure 6d). This reduction of S-phase cells was more evident after longer treatment (48 h) with higher concentrations of KSY (1500 or 2000 μg/ml) (Additional file 4: Figure S2).

### The toxicity assessment of in vivo KSY treatment in immunocompetent tumor-bearing mice

**Figure 7:**
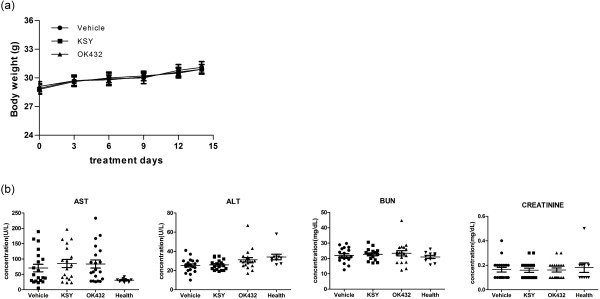
**KSY did not cause toxic effects in immunocompetent tumor-bearing mice.** The experimental protocol of bladder cancer cell (C3-B model in Figure 1) was performed. The body weights **(a)** and serum biochemical data **(b)** from various treatment groups were shown. n = 18-21 for each group. The serum data from healthy mice (n = 9-10) serves as reference values: AST: 30.9 ± 5.7 U/L; ALT: 34.1 ± 9.0 U/L; BUN: 21.2 ± 3.4 mg/dL; Creatinine: 0.2 ± 0.1 mg/dL.

The body weight, hepatic and renal function were measured in C3-B model. The serum levels of alanine aminotransferase and aspartate aminotransferase (AST) were measured as an index of hepatic function, and the levels of blood urea nitrogen (BUN) and creatinine served as an index of renal function. No difference in these parameters was observed between the KSY and control groups, demonstrating the safety of KSY administration in tumor-bearing mice under this treatment protocol (Figure 7a and b).

### The therapeutic and toxicity effects of KSY in a lung cancer model

**Figure 8:**
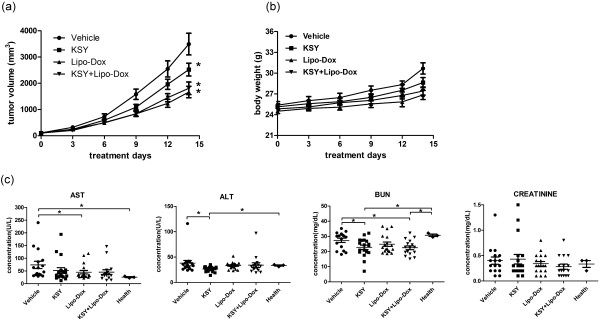
**The tumor-suppressive effects and toxicity assessments in a lung cancer model. (a)** The experimental protocol of lung cancer model (B6-L model in Figure 1) was performed and the tumor volumes were measured (n = 15-17 for each treatment group). **(b)** The body weights from all treatment groups are shown. **(c)** The serum biochemical data, from treatment groups or from healthy mice, were measured. The serum data from healthy mice (n = 3) serves as reference values: AST: 25.3 ± 3.5 U/L; ALT: 33.3 ± 2.1 U/L; BUN: 30.7 ± 1.4 mg/dL; Creatinine: 0.3 ± 0.1 mg/dL. *indicates *p* < 0.05 versus vehicle or healthy group.

To evaluate whether *in vivo* KSY treatment could suppress other type of malignant tumor, a syngeneic lung cancer mouse model was used (Figure 1, B6-L model). In addition, the combination treatment of KSY and doxorubicin was performed to assess the chemosensitizing effect of KSY. As shown in Figure 8a, the tumor volumes in mice treated with KSY, Lipo-Dox, and co-treatment were significantly reduced compared with the vehicle group, revealing the tumor suppression activity of KSY in this model. However, there was no difference on tumor growth between the combined treatment group and Lipo-Dox group.

The toxicity effects in B6-L model mice were also assessed. Compared to the vehicle group, KSY and co-treatment did not induce significant differences in body weights (Figure 8b) or in the serum levels of AST and creatinine (Figure 8c). These biochemical values were within the normal ranges described in the Mouse Phenome Database [28]. Therefore, KSY treatment along or combination with Lipo-Dox did not induce significant hepatic and renal toxicity in B6-L model mice.

## Discussion

KSY treatment delayed tumor growth in both immunocompetent and immunocompromised tumor-bearing mice (Figure 2), indicating that KSY could impair tumor growth with or without an intact immune system. The tumor-suppressive effects of KSY in immunocompromised hosts further validated that KSY had direct inhibitory effects on tumor cells. In tumor-bearing immunocompetent mice, neither the number of tumor infiltrated lymphocytes nor the anti-cancer cytokines were increased after KSY treatment (Figure 3). Collectively, the data showed that the potential anticancer activity of KSY acted mainly through the suppression of bladder cancer cells rather than the stimulation of anticancer immunity (Figures 2 and 3).

The immune cells in tumor microenvironment have been found to modulate tumor progression [29]. Induction of anticancer immunity is considered as a promising strategy for cancer therapy [30, 31]. Several ingredients of KSY have been previously demonstrated to exhibit immunomodulatory activity in pre-clinical studies [10–19]. Conversely, our data showed that KSY did not appear to induce immunity in these models and the anticancer activity of KSY is independent of the immune modulation (Figures 2 and 3). The discrepancy between these observations and previous reports regarding the immune-modulating effects may result from differences in the dosages, the experimental conditions used, the extraction methods or even the processing of KSY. Alternatively, possible synergistic or buffering interactions between these herbs may exist; thus, the pharmacological effects of KSY may be more complex and different from the effects of one single herb.

The inhibitory effects of KSY on cancer cells in vivo primarily resulted from the impairment of proliferation rather than the enhancement of apoptosis (Figures 4 and 5). Such growth-suppressive effects of KSY might be related to the increased expression of p53 and p21 (Figure 4). Both p53 and p21 are well-documented tumor suppressors and p21 is a known downstream gene of p53. The expression of p53 and p21 could be induced in response to various cellular stresses, leading to the down-regulation of cyclin-dependent kinases to impair cell cycle progression [32]. Although the induction of p21 and p53 has also been reported to promote apoptosis of cancer cells [33, 34], such correlation were not observed in the *in vivo* data (Figure 5). In line with the in vivo findings, direct treatment of bladder tumor cells with KSY led to cytostasis, in which both DNA synthesis and cell cycle progression were impaired (Figure 6). Based on aforementioned observation, growth suppression is the major anticancer effect of KSY, which was different from the immune-chemotherapeutic effects of OK432 [35] and the cytotoxic effects of Lipo-dox [36]. Multiple molecular mechanisms have been proposed to modulate the p53-controlled target gene selection and cell fate determination [37]. It remains to be clarified whether KSY treatment triggers any of these regulatory mechanisms to specifically promote p53-dependent growth inhibition.

In clinical trial, KSY administration had been proven to improve quality of life and hepatic function in Hepatitis B inactive carriers without causing major adverse effects [9]. In this study, KSY treatment at a dosage comparable to that used in clinical study induced neither hepatic injury or nephrotoxicity in bladder or lung cancer mouse models whether combination with chemotherapy or not (Figures 7 and 8). Qi-promoting herbal medicine, such as KSY in this study, is traditionally considered to function as adjuvant for chemotherapeutic drugs [38, 39]. Nevertheless, our data indicated that KSY did not enhance the therapeutic effect of chemotherapy drug doxorubicin (Figure 8). Since the combined treatment in our study was carried out in limited experimental conditions, the potential beneficial effects of KSY in combined with chemotherapy certainly merit further extensive investigation.

## Conclusions

In this study, the anticancer potential and the safety of KSY were demonstrated, and the possible anticancer mechanism of KSY was through the suppression of cancer cell proliferation, instead of immunomodulation effects. Until now, few studies have described the pharmacological activity of KSY, and this study is the first to reveal the potential tumor-suppressive effects of KSY using murine cancer models. In the future, more studies are warranted to further explore the therapeutic effect, action mechanism and herb-drug interactions of KSY with conventional cancer therapeutics.

## Electronic supplementary material

Additional file 1: **Supplemental method.** (PDF 55 KB)

Additional file 2: Table S1: List of primers used for q-RT-PCR. (PDF 26 KB)

Additional file 3: Figure S1: HPLC analysis of extracts from KSY and its constituted herbs. (PDF 236 KB)

Additional file 4: Figure S2: KSY treatment reduces BrdU-positive cells and interferes with cell cycle progression. (PDF 92 KB)

## Abbreviations

KSY: Kuan-Sin-Yin
C3-B: C3H/HeN mice inoculated with MBT-2 cancer cells
Nu-B: BALB/cAnN-Foxn1nu/Cr1Nar1 mice inoculated with MBT-2 cancer cells
B6-L: C57BL/6 inoculated with LLC-1 lung cancer cells
MTT: 3-(4,5-Dimethylthiazol-2-yl)-2,5-diphenyltetrazolium bromide
LPS: Lipopolysaccharide
PHA: Phytohemagglutinin
DAPI: 4’,6-diamidino-2-phenylindole
BrdU: 5-bromo-2’-deoxyuridine
ALT: Alanine aminotransferase
AST: Aspartate aminotransferase
BUN: Blood urea nitrogen.
