# Supplementary material for: Effects of Kuan-Sin-Yin decoction on immunomodulation and tumorigenesis in mouse tumor models
Source: BMC Complement Altern Med. 2014 Dec 15;14:488. doi: 10.1186/1472-6882-14-488 (PMC4301833; doi:10.1186/1472-6882-14-488)
Supplement: Supplementary file 1 — Additional file 1: Supplemental method. (PDF 55 KB) [file 12906_2014_2071_MOESM1_ESM.pdf]

# Additional file 1: supplemental method

## The HPLC analysis of KSY

The aqueous extract of KSY, extract of individual herb, and standard solution (calycosin-7-O-β-D-glucoside, liquiritinapioside, liquiritin, and glycyrrhizic acid) were pre-treated by ultrasonic agitation for 30 min and filtered through a 0.45 μm PTFE membrane filter. Subsequently, 20-30 microliter of each sample was respectively injected into a HPLC equipped with Waters 600C controller, a Waters 717 plus autosampler, a Waters 2996 photodiode array detector, and a 250 × 4.6 mm column with particle size 5 μm (Inertsil ph, GL Sciences, Inc., CA, USA). A 0.1 % phosphoric acid in water (A) and acetonitrile (B) was used as the mobile phase, the gradient elution was programmed as follows: 0–10 min, A/B:100/0→90/10; 10–30 min, A/B:90/10→80/20; 30–60 min, A/B:80/20→70/30; 60–100 min, A/B:70/30→40/60; 100–110 min, A/B:40/60→0/100;110–120 min, 100% B. Column temperature was maintained at room temperature. Analysis was performed at a flow rate of 0.8 mL/min with the detector wavelength set at 210nm - 400 nm.
