# Supplementary material for: Effects of Kuan-Sin-Yin decoction on immunomodulation and tumorigenesis in mouse tumor models
Source: BMC Complement Altern Med. 2014 Dec 15;14:488. doi: 10.1186/1472-6882-14-488 (PMC4301833; doi:10.1186/1472-6882-14-488)
Supplement: Supplementary file 2 — Additional file 2: Table S1: List of primers used for q-RT-PCR. (PDF 26 KB) [file 12906_2014_2071_MOESM2_ESM.pdf]

Additional file 2

Supplemental Table 1. List of primers used for q-RT-PCR

Gene Symbol	Sequence (5’ to 3’)
CD4 (mouse)	Sense CCC AAG CCT TCC ATA GAA ATC AA
	Anti-sense CCA AGT ATA GTC AAC ATG CTG GT
CD8 (mouse)	Sense CCG TTG ACC CGC TTT CTG T
	Anti-sense CGG CGT CCA TTT TCT TTG GAA
CD11b (mouse)	Sense ATG GAC GCT GAT GGC AAT ACC
	Anti-sense TCC CCA TTC ACG TCT CCCA
CD11c (mouse)	Sense CTG GGC CTG TCC CTT GCT
	Anti-sense ACA GTA GGA CCA CAA GCC AAC A
NK1.1 (mouse)	Sense TGG GAG GAA GGT CTA GTT GAT TG
	Anti-sense TCT CCT GAG ATA GCA GCA CAG
GAPDH (mouse)	Sense TGT GAT GGG TGT GAA CCA CGA G
	Anti-sense TGC TGT TGA AGT CGC AGG AGA C
