# Supplementary material for: Effects of Kuan-Sin-Yin decoction on immunomodulation and tumorigenesis in mouse tumor models
Source: BMC Complement Altern Med. 2014 Dec 15;14:488. doi: 10.1186/1472-6882-14-488 (PMC4301833; doi:10.1186/1472-6882-14-488)
Supplement: Supplementary file 3 — Additional file 3: Figure S1: HPLC analysis of extracts from KSY and its constituted herbs. (PDF 236 KB) [file 12906_2014_2071_MOESM3_ESM.pdf]

Additional file 3: Supplemental figure S1

(a)

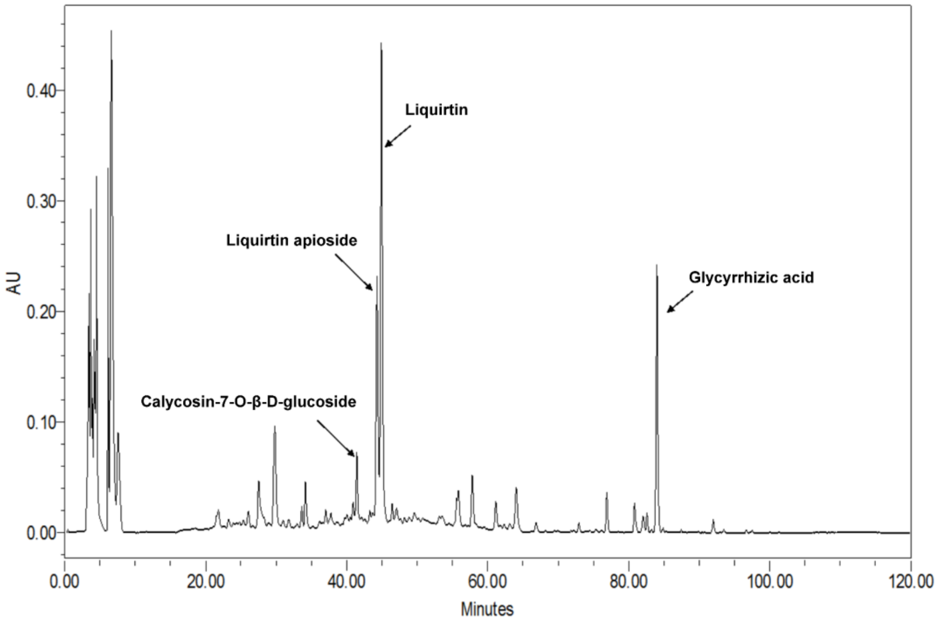

(b)

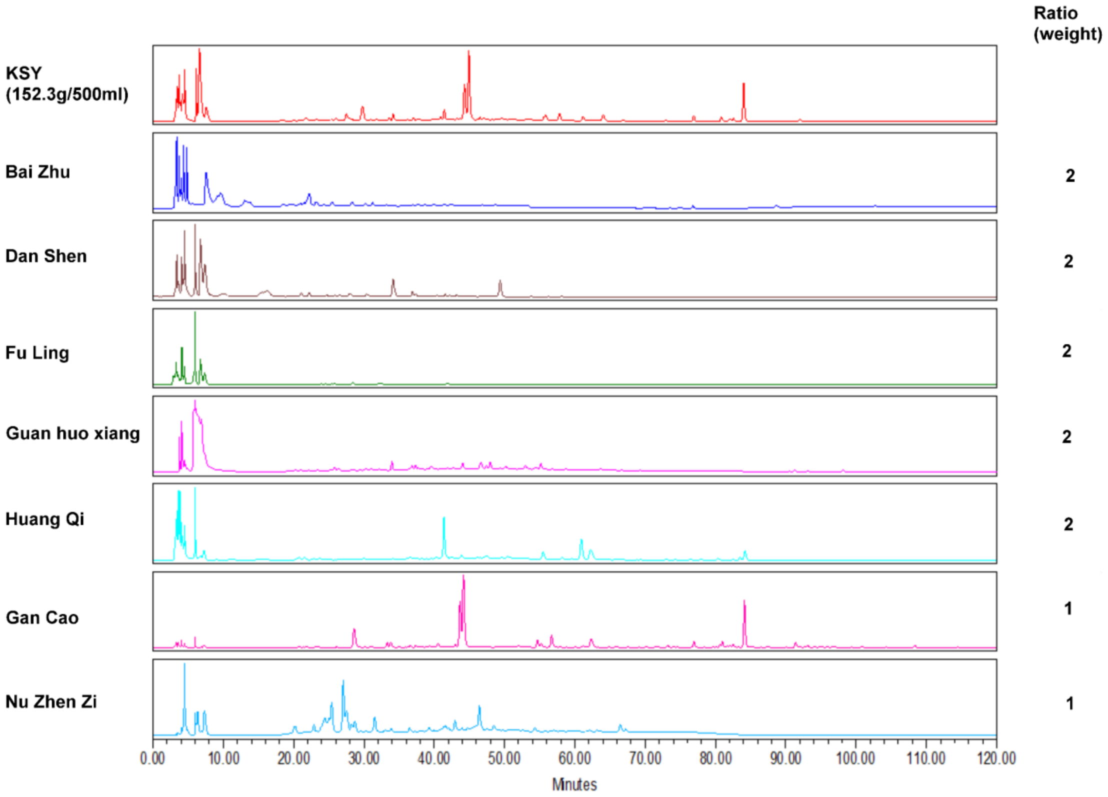

Supplemental figure 1. HPLC analysis of extracts from KSY and its constituted herbs

(a) The HPLC profile of KSY. Standard compounds (calycosin-7-O-β-D-glucoside, glycyrrhizicacid, liquiritin, liquiritinapioside) identified in KSY were marked. (b) Comparison of HPLC profiles between each herbal extract and KSY.
