# Supplementary material for: Effects of Kuan-Sin-Yin decoction on immunomodulation and tumorigenesis in mouse tumor models
Source: BMC Complement Altern Med. 2014 Dec 15;14:488. doi: 10.1186/1472-6882-14-488 (PMC4301833; doi:10.1186/1472-6882-14-488)
Supplement: Supplementary file 4 — Additional file 4: Figure S2: KSY treatment reduces BrdU-positive cells and interferes with cell cycle progression. (PDF 92 KB) [file 12906_2014_2071_MOESM4_ESM.pdf]

Additional file 4: Supplemental figure 2

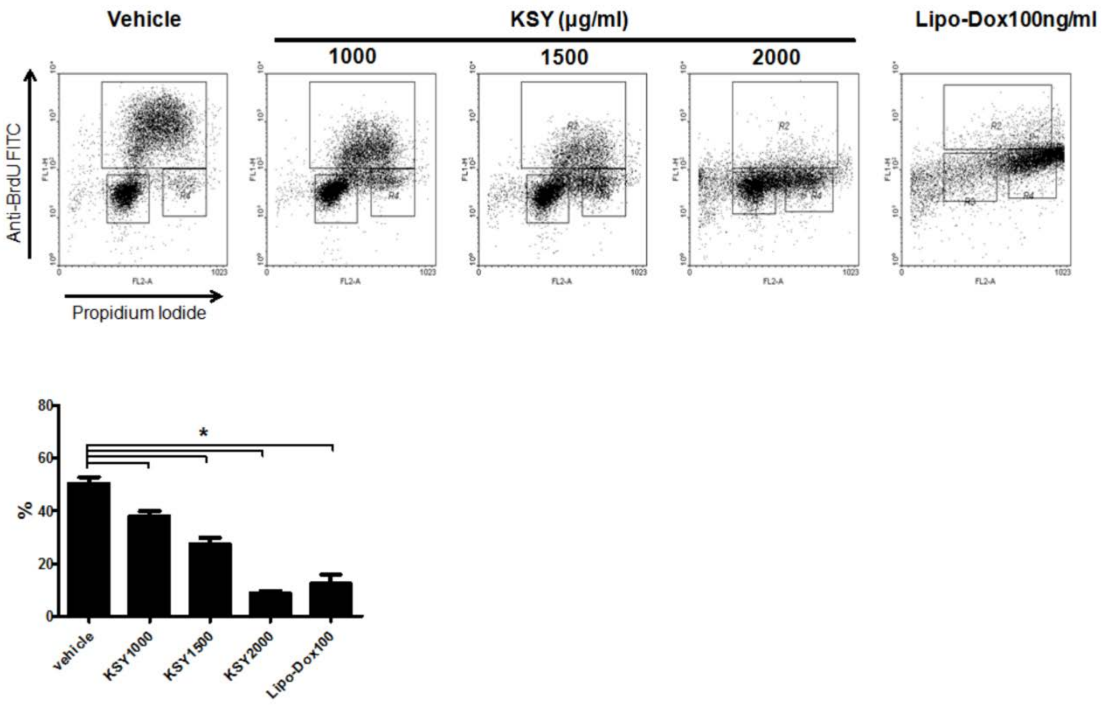

Supplemental figure 2. KSY treatment reduces BrdU-positive cells and interferes with cell cycle progression.

MBT-2 cells (1×10^5) were treated with different concentrations of KSY for 48 h, and then analyzed with BrdU/PI staining and flow cytometry. Representative FACS dot plots are shown in the upper panel, and DNA synthesis (S phase) was determined by quantifying the BrdU-positive cells in the dot plot. Quantification data of BrdU-positive cells are shown in the lower panel. * indicates p<0.05 versus the vehicle group. Three independent experiments were performed.
